# Editorial: Antimicrobial and Anticancer Peptides

**DOI:** 10.3389/fchem.2018.00013

**Published:** 2018-02-06

**Authors:** Neil M. O'Brien-Simpson, Ralf Hoffmann, C. S. Brian Chia, John D. Wade

**Affiliations:** ^1^Oral Health Cooperative Research Centre, Melbourne Dental School, University of Melbourne, Parkville, VIC, Australia; ^2^Bio21 Institute, University of Melbourne, Parkville, VIC, Australia; ^3^Faculty of Chemistry and Mineralogy, Institute of Bioanalytical Chemistry, Universität Leipzig, Leipzig, Germany; ^4^Center for Biotechnology and Biomedicine, Universität Leipzig, Leipzig, Germany; ^5^Experimental Therapeutics Centre, Agency for Science, Technology and Research (A^*^STAR), Singapore, Singapore; ^6^Florey Institute of Neuroscience and Mental Health, University of Melbourne, Parkville, VIC, Australia; ^7^School of Chemistry, University of Melbourne, Parkville, VIC, Australia

**Keywords:** antimmicrobial, anticancer, peptides, cancer, antibiotic resistance, bacteria, microbes, peptide chemistry

The World Health Organization (WHO) in their 2014 report describe cancer as “one of the leading causes of morbidity and mortality worldwide” and is the second leading cause of death globally (Stewart et al., 2014). In the same year, WHO recognized that antimicrobial resistance (AMR) as “a key global health challenge” and, if not addressed, estimated the annual number of deaths due to antibiotic-resistant bacteria could reach 10 million by 2050, exceeding that of cancer by 1.2 million (O'Neill, 2014; World-Health-Organisation, 2014). On the face of it, these reports may seem to be disconnected, however, surgery and chemotherapy, are two major therapies in cancer treatment that rely on the use of antibiotics/antimicrobials to prevent microbial infection post-surgery and when chemotherapy suppresses the immune system. Thus, the impact of a post-antibiotic era has far more reaching consequences than our ability to treat infections which will significantly impact the delivery and management of health care as a whole. According to WHO and the UK Prime Minister's Office reports (O'Neill, 2014; Stewart et al., 2014; World-Health-Organisation, 2014, 2017), there are urgent calls for more targeted approaches for cancer treatment and to address the global emergence of AMR. For both cancer and multi-drug resistant microbes, peptides are deemed as the next generation antimicrobial materials and strategies are being developed to design cell specific peptide-based therapeutics. In this Research Topic we present a salient collection of original research and review articles that show how multidisciplinary approaches to antimicrobial resistance and cancer are leading to the discovery and production of novel materials with specificity toward bacteria and/or cancer cells.

Intriguingly, it has been shown that cationic antimicrobial peptides (AMPs) have anticancer properties which may be due to both bacteria and cancer cell membranes having a net negative charge. This dual nature of peptides of having antimicrobial and anticancer properties is explored in the review by Felício et al. The discovery of bioactive peptides is an essential part of medicinal and peptide chemistry research and Díaz-Gómez et al. highlight this in the review article on anticancer peptides that have been discovered and isolated from maize. The importance of bioactive peptide discovery is further highlighted by Oyama et al. who designed an AMP that has potent antibacterial activity against *Enterococcus faecalis* that was identified from a rumen metagenomics study. A significant advantage of peptides as therapeutics is that they are straightforwardly modified by solid and solution phase chemical techniques, facilitating structure-relationship (SAR) studies on newly-discovered peptides. Insect defensins are a class of AMPs that have drawn considerable attention and SAR studies are being used to define bioactive and biotoxic sequences with the purpose of designing novel cysteine-rich highly effective AMPs (Koehbach).

Altering specific peptide residues with D- and unnatural amino acids can significantly impact on biological activity as shown by Li et al. on the activity of the AMP Chex1-Arg20. An approach taken by Mardirossian et al. is the synthesis of an all D-enantiomer of BMAP18 which showed improved *in vitro* protease stability and then demonstrated *in vivo* that other factors inhibited activity and these need to be considered in AMP design. In the review by Casciaro et al. how the use of D- and unnatural amino acids and conjugation to nanoparticles is impact on an AMPs bio-stability, cytotoxicity and delivery of the AMP to a specific target site is assessed. The use of stable isotope-labeled peptides of apidaecin analogs, pharmaocokinetics, and mass spectroscopy revealed how apidaecin AMPs were effective *in vivo* and demonstrates how these *in vivo* methods can be applied to other AMPs (Schmidt).

AMPs can be used as surface coatings on medical devices to prevent microbial colonization as reviewed by Riool et al. With an emphasis on how AMPs are conjugated on to biomedical devices and how effective they are López-Pérez et al. describe how SPOT synthesis can be employed to screen and optimize the activity of surface-bound AMPs. Colonization of surfaces by bacteria typically leads to the development of a biofilm and Grassi et al. demonstrate that AMP activity against biofilms can be enhanced by co-administration of adjuvant-like molecules that aid membrane disruption. Many AMPs exert their action directly on the cytoplasmic membrane of bacteria and various models of their mechanism of action have been proposed. However, Zeth and Sancho-Vaello question these models as they show that two well-studied AMPs, LL-37 and dermcidin, deviate from the traditional models of membrane-disruption. The novel nature of AMP modes of action differ significantly from traditional antibiotics and drugs and yet AMPs fall under the same drug approval regulations, hindering AMP development as highlighted by Otvos in a perspective article (Otvos). Finally, the complex nature of the mode of action of AMPs is emphasized by Del Cogliano et al. who show that cationic AMPs are able to inactivate Shiga toxin-encoding bacteriophages and thus reduce *Escherichia coli* virulence.

We believe that the Antimicrobial and Anticancer Peptide Research Topic exemplifies the multidisciplinary nature of peptide research and that the advancement of therapeutics that target cancer and/or microbes requires an interconnected research strategy as exemplified in this body of work. It has the objective of stimulating new avenues of thinking, approaches, and collaboration in tackling current and forthcoming cancer and antimicrobial resistance health threats.

## Author contributions

All authors listed have made a substantial, direct, and intellectual contribution to the work, and approved it for publication.

### Conflict of interest statement

The authors declare that the research was conducted in the absence of any commercial or financial relationships that could be construed as a potential conflict of interest.
